# A New Approach to Control the Enigmatic Activity of Aldose Reductase

**DOI:** 10.1371/journal.pone.0074076

**Published:** 2013-09-03

**Authors:** Antonella Del-Corso, Francesco Balestri, Elisa Di Bugno, Roberta Moschini, Mario Cappiello, Stefania Sartini, Concettina La-Motta, Federico Da-Settimo, Umberto Mura

**Affiliations:** 1 Biochemistry Unit at the Department of Biology, University of Pisa, Pisa, Italy; 2 Department of Pharmaceutical Sciences, University of Pisa, Pisa, Italy; University of Bologna & Italian Institute of Technology, Italy

## Abstract

Aldose reductase (AR) is an NADPH-dependent reductase, which acts on a variety of hydrophilic as well as hydrophobic aldehydes. It is currently defined as the first enzyme in the so-called polyol pathway, in which glucose is transformed into sorbitol by AR and then to fructose by an NAD^+^-dependent dehydrogenase. An exaggerated flux of glucose through the polyol pathway (as can occur in diabetes) with the subsequent accumulation of sorbitol, was originally proposed as the basic event in the aethiology of secondary diabetic complications. For decades this has meant targeting the enzyme for a specific and strong inhibition. However, the ability of AR to reduce toxic alkenals and alkanals, which are products of oxidative stress, poses the question of whether AR might be better classified as a detoxifying enzyme, thus raising doubts as to the unequivocal advantages of inhibiting the enzyme. This paper provides evidence of the possibility for an effective intervention on AR activity through an intra-site differential inhibition. Examples of a new generation of aldose reductase “differential” inhibitors (ARDIs) are presented, which can preferentially inhibit the reduction of either hydrophilic or hydrophobic substrates. Some selected inhibitors are shown to preferentially inhibit enzyme activity on glucose or glyceraldehyde and 3-glutathionyl-4-hydroxy-nonanal, but are less effective in reducing 4-hydroxy-2-nonenal. We question the efficacy of D, L-glyceraldehyde, the substrate commonly used in *in vitro* inhibition AR studies, as an *in vitro* reference AR substrate when the aim of the investigation is to impair glucose reduction.

## Introduction

Aldose reductase (AR) is an NADPH-dependent [1] aldo-keto reductase (EC 1.1.1.21) that catalyzes the reduction of a variety of hydrophobic as well as hydrophilic aldehydes (for reviews, see 2,3). The enzyme is considered as part of the so-called polyol pathway in which glucose is first reduced by AR to sorbitol, which is then oxidized to fructose by a NAD^+^ dependent sorbitol dehydrogenase [4]. An increased flux of glucose through the polyol pathway in hyperglycemic conditions has been considered to cause tissue damage through different mechanisms, including an osmotic imbalance due to sorbitol accumulation [5], an imbalance of the pyridine nucleotide redox status, which decreases the antioxidant cell ability [6], and an increase in the advanced glycated end products [7-9]. All these cell-damaging processes can cause diabetic complications, such as nephropathies, retinopathies, peripheral neuropathies and cataract. Consequently, AR has been considered as a target enzyme to develop drugs that act as AR inhibitors (ARIs), which are thus able to prevent the onset of diabetic complications and to control their evolution.

Recently, AR has been shown to be involved in ischemic and inflammatory processes [10-12] and to be overexpressed in some types of cancer [10,13]. This led to the increased interest in ARIs as anti-inflammatory agents [14]. Over the last three or four decades a number of ARIs have been discovered and then proposed as potential therapeutic tools. Despite the in vitro efficiency of ARIs, their use as drugs to antagonize diabetic complications has not been very successful (to the best of our knowledge India and Japan are the only countries where an Epalrestat-based drug is distributed). This is possibly because of an insufficient bioavailability [15,16] and/or a possible modulation in the AR susceptibility to inhibition exerted by S-thiolation phenomena [17-20]. Moreover, some ARIs have been withdrawn due to the appearance of severe secondary effects in preclinical and/or clinical trials [21,22]. These adverse effects may be related to the impairment of some AR functions upon ARI treatment. In fact one of the functions of AR is its ability to reduce toxic aldehydes, such as 4-hydroxy-2,3-nonenal (HNE), which are end products of lipid peroxidation [23], and whose cytotoxicity appears to be lower when they have been reduced. In addition, the ability of AR to reduce the glutathionyl-HNE adduct (GS-HNE) [24] represents a link between AR activity and the cell response to the oxidative signaling cascade [14,25]. The enzyme may also act as an osmoregulatory device [26,27] and plays an important role in the synthesis of fructose [4], tetrahydrobiopterin [28,29] and in the metabolism of corticosteroids [30-32].

All these aspects raise doubt for an *a priori* overall advantage in inhibiting the enzyme.

The possibility of selectively intervening on the enzyme’s catalytic action on specific substrates, such as glucose, is a clear benefit as it leaves the reduction of damaging molecules such as HNE unaffected or partially affected. These aldose reductase differential inhibitors (ARDIs) have the potential to target AR in strict relation to the substrate that the enzyme is working on. This means that damaging events (i.e. sorbitol and GS-DHN generation) could be blocked (fully or partially) without affecting the detoxification ability of the enzyme (i.e. HNE reduction).

The fact that hydrophilic molecules, such as GAL, glycol aldehyde or L-threose, and hydrophobic molecules, such as HNE, are similarly effective as AR substrates [33-35], suggests a rather poor selectivity of the enzyme, apparently permissive to the entrance of any kind of aldehydic substrate. However looking inside the same class of hydrophobic [34] as well as hydrophilic [33,36] molecules, it appears that AR is not simply a permissive enzyme, being able to discriminate different substrates among the same class. In any case, the ability of sugar molecules and hydrophobic aldehydes to interact with AR with the same or similar efficiency, would suggest that these molecules may interact with the enzyme following different interactive pathways.

The peculiar ability of AR to intervene both on hydrophilic and hydrophobic substrates without being a permissive enzyme opens the possibility to identify ARDIs to be developed as possible useful instruments to modulate AR activity. The possibility of inhibiting AR while acting on aldoses but not on toxic aldehydes, previously recommended as a valuable task to be reached [37], was strengthened through an in silico approach [38] in which, considering an inhibitor-inducible cavity region located close to the active AR site [39], a number of AR inhibitory molecules were designed, which still need to be tested in terms of how well they are able to differentially inhibit the enzyme.

In this paper evidence is presented for an effective intervention on AR activity through an intra-site differential inhibition. Our data, which represent the results of what we believe is the first experimental attempt to use ARDIs in AR inhibition studies, suggest that searching for ARDIs may be a suitable approach.

## Materials and Methods

### Materials

NADPH, D, L-glyceraldehyde (GAL), D-glucose, dithiothreitol (DTT), sodium EDTA, were purchased from Sigma-Aldrich Italy (Milano, Italy); HNE dimethylacetal was purchased from Enzo Life Science Inc. (New York, USA) and the free aldehyde was obtained with the addition of 1 mM HCl; GS-HNE came from Cayman Europe (Tallin, Estonia). All inorganic chemicals (from BDH, London, UK) were of reagent grade.

### AR inhibitors

Molecules tested as AR inhibitors are identified by code numbers in bold. The structures of the compounds tested as AR inhibitors are reported in Tables S1 and S2.

The pyrido(1,2-*a*) pyrimidinones **1-8** [40], oxadiazole **9** [41], naphtho(1,2-*d*) isothiazole**10** [42], (1,2,4) triazino(4,3-*a*) benzimidazoles **11,12** and the benzimidazole **13** [43], benzisotiazoles 14-16 [44], and **17** [45] were synthesized as described in the references. The pyrazolo(1,5-*a*)pyrimidines **18-20** and amides **21**-**25** were synthesized as reported in the Protocol S1. D gluconamide (26), D-lactamide (27), Quercetin (28) and Sorbinil (29) came from Sigma-Aldrich Italy. Epalrestat (30) was from Haorui Pharma-Chem Inc., NJ, USA.

### AR assay and purification

If not otherwise specified AR activity was determined at 37°C using 4.7 mM D, L-GAL as a substrate in 0.25 M sodium phosphate buffer pH 6.8 containing 0.38 M ammonium sulfate, 0.5 mM EDTA, and 0.2 mM NADPH. AR was purified from bovine lens to electrophoretic homogeneity as described [46]. The final enzyme preparation displayed a specific activity of 1.2 U/mg. One unit of enzyme activity is the amount of enzyme that catalyzes the oxidation of 1 µmol of NADPH/min under the above-mentioned conditions. The *k*_*cat*_ values were obtained on the basis of a molecular weight of AR of 34 KDa. The purified enzyme was stored at 4°C in a 10 mM sodium phosphate buffer pH 7.0 supplemented by 2 mM dithiothreitol and was extensively dialyzed against a 10 mM sodium phosphate buffer pH 7.0 before use.

The nominal concentrations of HNE and GS-HNE solutions used as substrates of AR were determined through a colorimetric assay [47]. These values were confirmed by the total decrease in absorbance at 340 nm measured, in the presence of 0.2 mM NADPH, when HNE and GS-HNE were incubated in the presence of 8 and 150 mU of AR, respectively (Figure S1). When testing inhibitors that require DMSO to be solubilized, the same concentration of DMSO (0.7% v/v), was used in the assays both in the presence and absence of the inhibitors.

### Other methods

Protein concentration was determined according to Bradford [48] using bovine serum albumin as the standard.

## Results and Discussion

**Figure 1:**
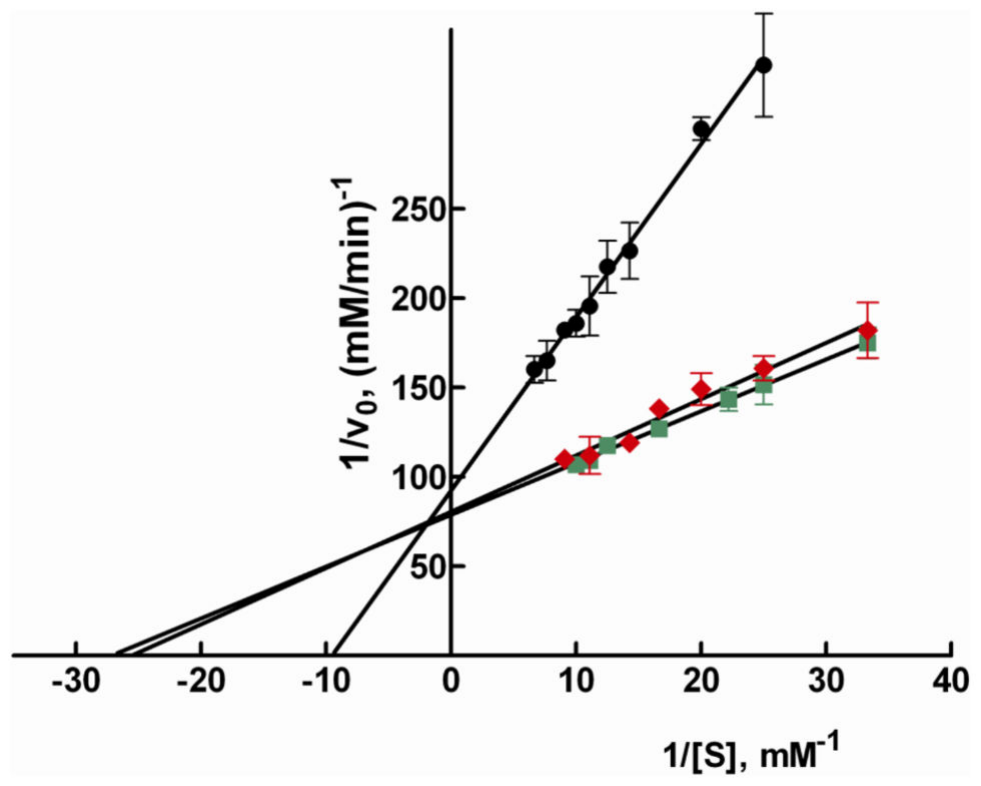
Double reciprocal plots for reduction of GAL, HNE and GS-HNE by bovine lens AR. Double reciprocal plots of initial rate measurements using of GAL (diamonds), HNE (squares) and GS-HNE (circles) as substrates. The assays were performed in standard conditions using 8 mU of purified AR. Error bars represent the standard deviations from at least three independent measurements.

GAL and HNE used as substrates for AR displayed a very similar kinetic behaviour. As shown in Figure 1 the double reciprocal plots for the reduction of the two substrates by the same purified enzyme preparation are almost superimposable showing *k*_*cat*_ and K_M_ for GAL and HNE of 47 ± 3 and 48 ±1 min^-1^ and 39 ± 4 and 37 ± 1 µM, respectively.

The K_M_ for GAL is in line with previously reported data for AR from different sources, including the bovine lens enzyme [24,49-53]. Similarly, the K_M_ measured for HNE is reasonably close to the one measured on human skeletal muscle (22 µM) and human placental recombinant (28 ± 9 µM) enzymes [34,35]. Nevertheless the K_M_ measured for HNE appears approximately five fold higher than the values reported for the AR from bovine tissues, namely the lens (8.8±0.9 µM) and the heart (7 ±2 µM) [24,52]. In addition to the more acidic assay conditions (pH 6.0 rather than 6.8) adopted in previous measurements, which, however, were not responsible for the observed difference (Figure S2), it may be worth considering the potential of HNE to inactivate AR [52,54]. In fact, even though NADP^+^ has been reported to exert protection [52,54], enzyme inactivation may still occur to some extent while the enzyme is acting, especially at relatively high concentrations of HNE. This is evident by applying the Selwyn test [55] to the AR catalyzed reaction performed both at different concentrations of HNE (from 50 to 220 µM) and at different concentrations of the enzyme (from 3.5 to 8.8 mU) (Figure S3). The underestimation of the reaction rate occurring at high substrate concentrations may lead to an overestimation of the apparent affinity between HNE and AR. Thus, in this study the assays were performed in the presence of 8 mU of AR using HNE concentrations that were never above 0.11 mM, i.e. a safe limit according to the Selwyn test. The insertion of a hydrophilic moiety into HNE, as it occurs for the GS-HNE adduct, determined, differently from that observed for other alkanals and alkenals [51], a modest decrease of the *k*_*cat*_ (41 ± 2 min^-1^) with approximately a 3 fold increase of the K_M_ (105 ± 6µM) with respect to the parameters measured for HNE (Figure 1). However, in the case of GS-HNE, a stable intramolecular hemiacetal may be formed and this could reduce the concentration of the free carbonyl with an increase in the apparent K_M_. The occurrence of the stable hemiacetal is consistent with the higher amount of AR required for GS-HNE titration with respect to HNE (Figure S1). Concerning the *k*_cat_ value, it appears only slightly changed compared to HNE as a result of the insertion of the glutathionyl moiety into HNE. Note that the value of K_M_ for GS-HNE was approximately three fold higher than the previously reported result for the bovine lens enzyme [24].

**Figure 2:**
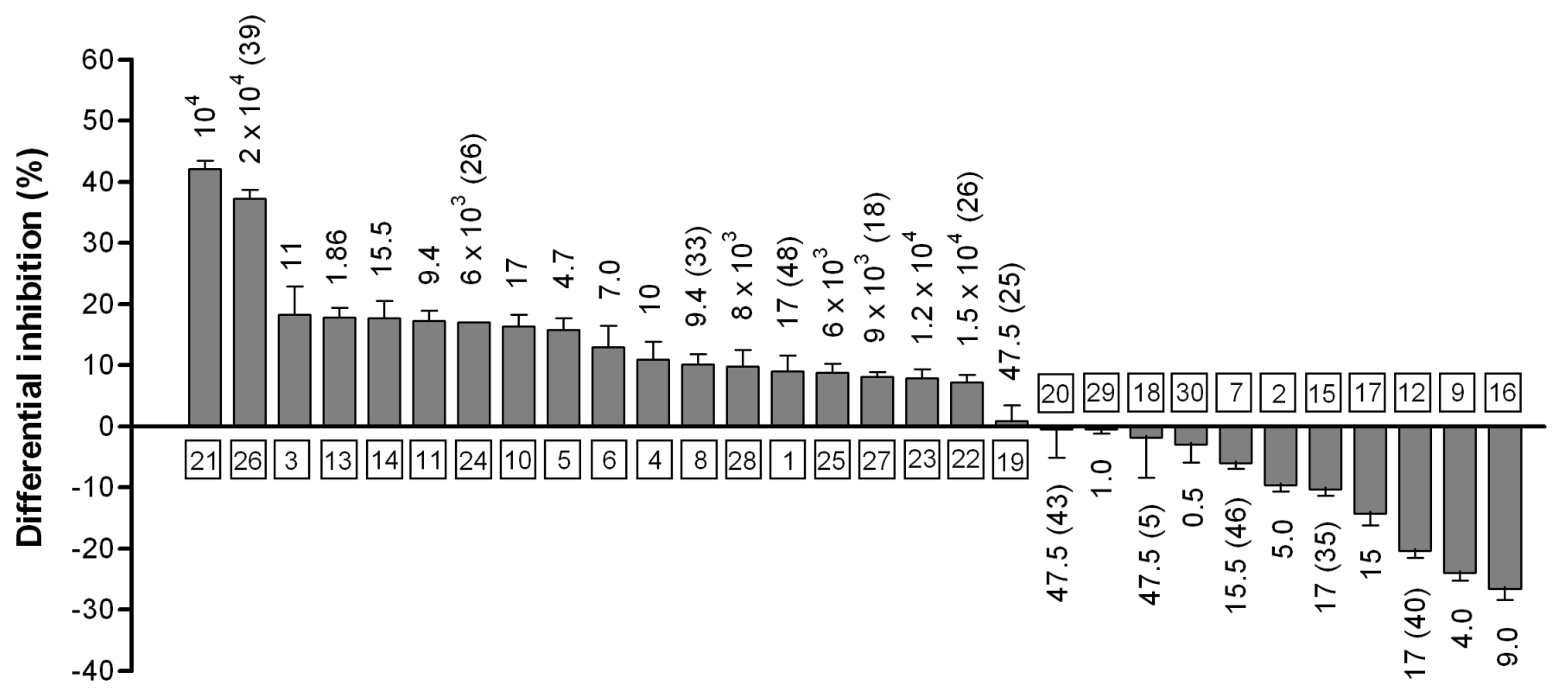
Differences in inhibition of AR depending on the nature of the substrate. Bars indicate the difference between the percentage inhibition observed using 0.11 mM GAL as substrate and the percentage inhibition using 0.11 mM HNE (differential inhibition). The difference was calculated at the concentration of the inhibitor leading to a 50% inhibition of the reaction more sensitive to the inhibitor (i.e. the reduction of GAL for compounds displaying a positive differential inhibition and the reduction HNE for compounds displaying a negative differential inhibition) or at the maximal concentration tested if 50% inhibition was not reached. Numbers on the bars refer either to the inhibitor concentration (µM) leading to 50% inhibition or to the maximal concentration (µM) tested together with the corresponding percentage inhibition (in brackets) observed with the substrate that is more sensitive to the inhibitor. Framed numbers (1 to 30) refer to the compounds tested as inhibitors.

The similarity of the kinetic parameters (K_M_ and *k*_*cat*_) displayed by GAL and HNE represents an ideal condition to compare the ability of different molecules to behave as ARDIs. Thus, the susceptibility of AR to the inhibition exerted by a series of compounds (including some well-known ARIs) was tested at various inhibitor concentrations, using both GAL and HNE as substrates. The results of this screening (Figure 2) are expressed as ”GAL/HNE differential inhibition”, i.e. the difference between the percentage inhibition observed using GAL as a substrate and the percentage inhibition observed using HNE as a substrate. This differential inhibition was calculated at the concentration of the inhibitor leading to 50% inhibition or, when 50% inhibition was not reached, at the maximal concentration tested. The data indicate that the purified bovine lens AR may vary in susceptibility to inhibition depending on the substrate the enzyme is working on. This is a case where there is a preferential inhibition of both GAL and HNE reduction, but also where no preferential inhibitory action against the reduction of a specific substrate takes place. Thus, differential inhibition values are observed, ranging from approximately +40% (i.e. HNE reduction is favored), exerted by GAM and D-gluconamide (compounds **21** and **26**, respectively), to -25% (i.e. GAL reduction is favored), exerted by compound **16**. Despite the number of compounds tested, it is difficult at this point to find a relationship between structure and ARDI behavior, mainly because of the structural heterogeneity as well as of the wide range of inhibitory power of the compounds. In this regard, the available PDB data of AR structures co-crystallized with very strong inhibitors are, as such, less helpful in finding special structural requirements for ARDIs than one would expect. In fact, they are lacking of the most relevant information concerning the reciprocal influence between the substrate and the inhibitor, both interacting with the enzyme.

The not permissive feature of AR, mentioned above for substrates (see Introduction), appears to be confirmed for inhibitors of the same class as well. In fact, starting with GAM, a variety of differential inhibitory effects was observed for different aldonamides (compounds **21**-**27**). Indeed, a significantly lower value of ”GAL/HNE differential inhibition” was observed (approximately 7%) when L-glyceramide (compound **22**) was used instead of the D-enantiomer (compound **22**, Figure 2).

**Figure 3:**
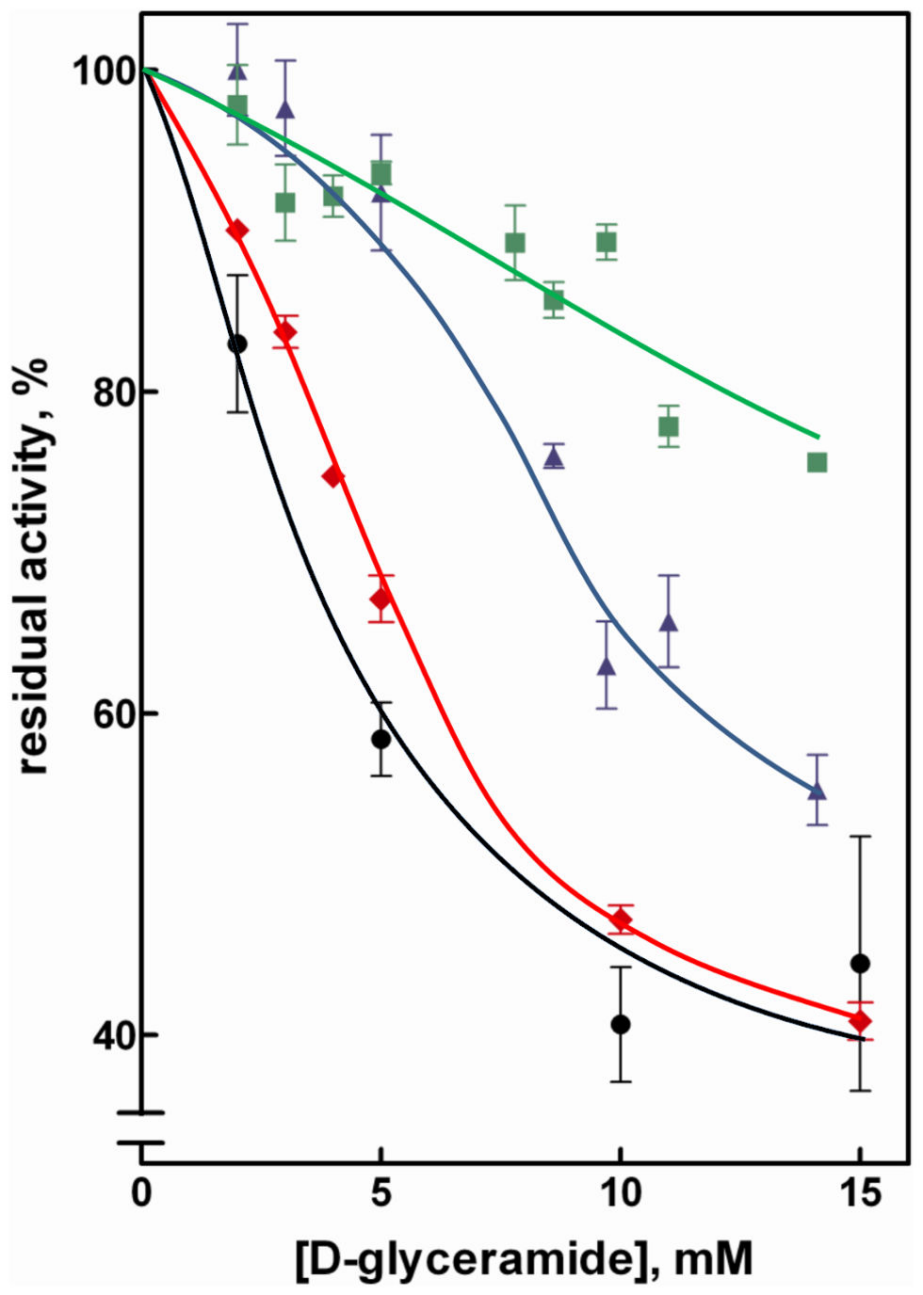
Effect of D-glyceramide as differential AR inhibitor. Percentage residual activity was determined at various inhibitor concentrations using the following substrates: 0.11 mM of HNE (squares), GAL (diamonds), GS-HNE (circles) or 7 mM D-glucose (triangles). Error bars represent the standard deviations from at least three independent measurements.

Similar to what was observed for GAL, the reduction of GS-HNE was also inhibited by GAM (IC_50_ of approximately 8 mM) more efficiently than the HNE reduction (Figure 3). In the case of glucose a differential inhibition glucose/HNE was observed only at rather high concentrations of GAM (Figure 3).

However, the kinetic behavior of both glucose and GS-HNE is very different from HNE (and consequently from GAL), which must be taken into account when directly comparing the differential inhibition data. In the case of glucose, for instance, the concentration used in the assay (7 mM), which is the blood concentration occurring in moderate hyperglycemic conditions, led to sub-saturating conditions considering the rather high K_M_ value of AR for glucose (35 to 212 mM) [33,49,50,56-58]. A similar situation may also take place with GS-HNE, which was assayed at a substrate concentration very close to the K_M_ value.

These data clearly show the potential of a specific AR intra-site differential inhibition. However, considering the differential inhibition values and the inhibitor concentrations required for GAM to exert inhibition (IC_50_ of approximately 10 mM with GAL as substrate), we failed to target high differential intra-site specificity and high inhibitory power at the same time.

**Figure 4:**
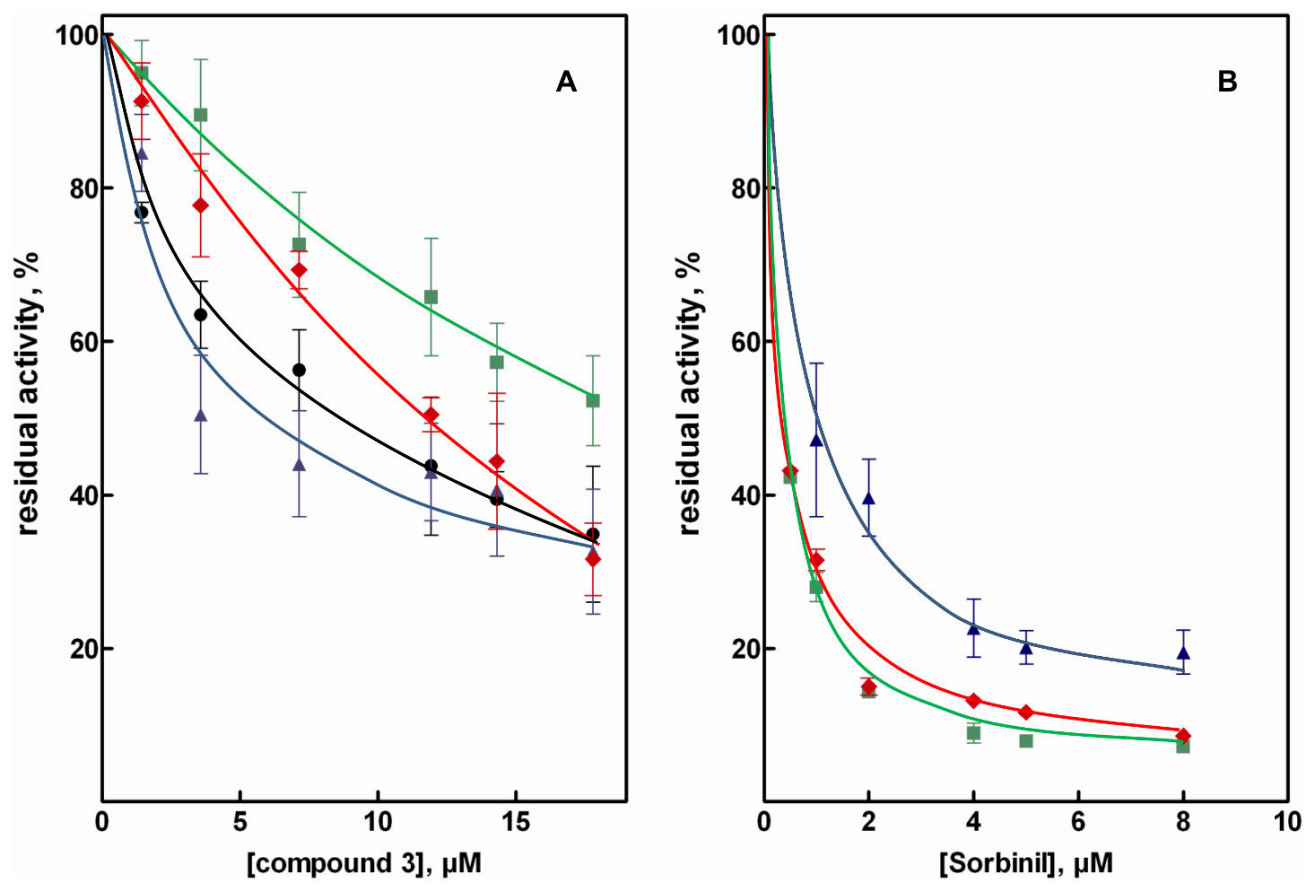
Effect of some ARIs on the AR-catalyzed reduction of different substrates. Percentage residual activity was determined at the indicated inhibitor concentrations using the following as substrate: 0.11 mM of HNE (squares), GAL (diamonds), GS-HNE (circles) or 7 mM D-glucose (triangles). *Panel a* and *b* refer to compound **3** and Sorbinil, respectively. Error bars represent the standard deviations from at least three completely independent measurements including different enzyme preparations.

The screening of molecules as potential ARDIs reported in Figure 2 highlights various critical issues. The first concerns the absolute values of inhibition exerted by the inhibitor on the reduction of HNE. In this regard, compound **3** can be taken as an example. Figure 4A reports the inhibition curves of compound **3**, obtained using GAL, HNE and glucose as substrates. This molecule exhibited a GAL/HNE differential inhibition of approximately 15%, a value that slightly increased when glucose was used as a substrate rather than GAL. However, this molecule cannot be defined as an ARDI. In fact it inhibits the AR catalyzed reduction of all the tested substrates, including HNE. In this respect compound **3** behaves, though less efficiently, like the classical ARI Sorbinil (compound 29), whose negligible GAL/HNE differential inhibition (Figure 2) was accompanied by its potent inhibitory action on all the tested substrates (Figure 4B).

Our study also highlights the fact that simply screening AR inhibitors using GAL as substrate might be not sufficient to select molecules, not only as potential ARDIs, but also as ARIs, especially when the aim of the investigation is drug development. Such screening is unavoidable as a preliminary step before an in depth kinetic analysis of the selected molecules, and is usually performed at rather high GAL concentrations [59-62]. This leads to an underestimation of the effectiveness of molecules which display a competitive or mixed noncompetitive type of inhibition, especially, in the latter case, when the component of the competitive action is dominant. To overcome this limitation, when screening for the selection of a potential inhibitor, it might be worth using glucose and possibly GS-HNE as substrates. In fact, unlike GAL or HNE, the rather high K_M_ values of glucose, would enable the measurement of AR activity in sub-saturating conditions without the possible limitations in the assay performance due to low substrate levels, as might occur with GAL and HNE. It is also evident that the use of glucose alone may be inadequate for inhibition screening since an underestimation of the effect of potential inhibitors with a marked prevalence of uncompetitive action may occur. This might be the case, for instance, of D-gluconamide (Figure 2) which, like GAM, exerts a significant GAL/HNE differential inhibition, but appears to have no effect on glucose reduction (data not shown).

**Figure 5:**
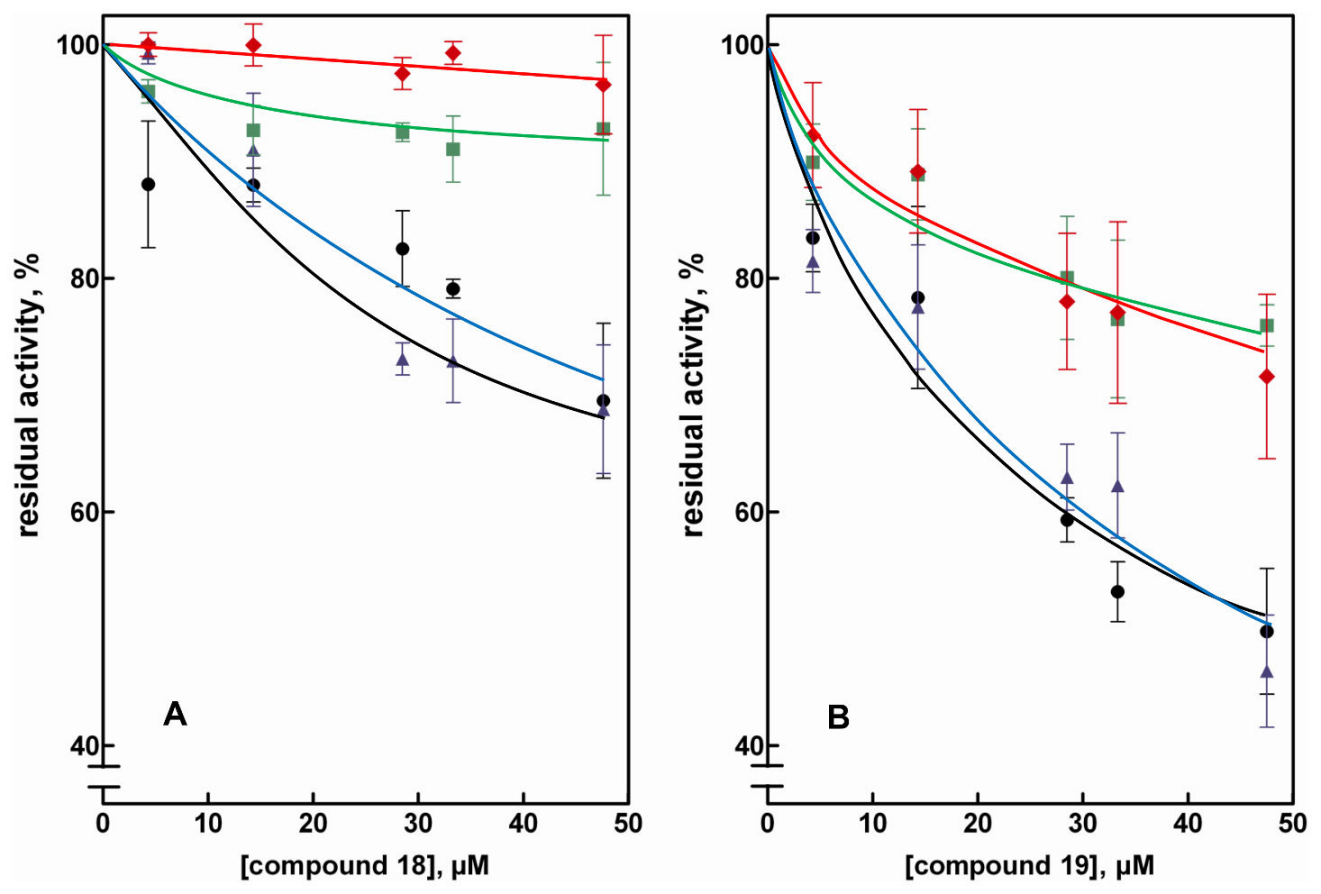
Effect of compounds acting as differential AR inhibitors. Percentage residual activity was determined at various inhibitor concentrations using the following substrates: 0.11 mM HNE (squares), GAL (diamonds), GS-HNE (circles) or 7 mM D-glucose (triangles). *Panels a* and *b* refer to compounds **18** and **19**, respectively. Error bars represent the standard deviations from at least three completely independent measurements including different enzyme preparations.

The problem of underestimating the possible differential inhibition ability of molecules is illustrated in Figure 5, which reports the inhibition of the AR-dependent reduction of GAL, HNE, D-glucose and GS-HNE by compounds **18** and **19**. Both compounds, which exert (see also Figure 2) an almost negligible GAL/HNE differential inhibition, inhibit the reduction of glucose more efficiently than the reduction of HNE with a glucose/HNE differential inhibition (evaluated at the highest inhibitor concentration tested) of 24 ± 8 and 30 ± 5% for compound **18**, and compound **19**, respectively. The observed effect of compound **18** on glucose reduction can be ascribed to a competitive inhibition, which is not revealed for both GAL and HNE despite lowering the concentration of these substrates to approximately the K_M_ values (Figures S4 and S5). For compound **19** the inhibitory action on glucose reduction is also described by an inhibition model where the competitive action dominates. The effects of compound **19** on the reduction of GAL and, more distinctly, of HNE, are different and are described by mixed and uncompetitive types of inhibition, respectively. These data clearly indicate how the same inhibitor may interact differentially with the enzyme depending on the substrate used. Taking into account the absolute concentrations at which these compounds are effective (μM range), they appear to have the potential to be developed as effective ARDIs.

Finally, as observed for GAM (Figure 3), both compounds **18** and **19** are also able to inhibit, to a different extent, the AR catalyzed reduction of GS-HNE (Figure 5). In this case, however, it is not easy to interpret the results in terms of the inhibition model. This difficulty may be related to the special structural features of GS-HNE, in which distinct hydrophilic and hydrophobic moieties coexist. The presence of cyclic hemiacetal stereo forms is also particular to this molecule, which makes it difficult to clearly define the univocal assay conditions in terms of substrate concentration.

In any event a differential inhibition between GS-HNE and HNE is clearly possible. Considering the proposed action of the GS-DHN (the reduction product of GS-HNE) as a pro-inflammatory signal [14,25], we believe that these results should be taken into consideration when searching for ARDIs with anti-inflammatory activities.

## Conclusions

We believe that the evidence presented here opens the way for a new strategic approach to AR inhibition. The focus of this work is not only on the features of potential ARDIs, but also on the type of experimental approach to be adopted in looking for ARIs. Indeed, by extending the same arguments used to predict the potential of a differential inhibition between substrates of different classes to different substrates of the same class (i.e. aldoses), our results strongly suggest that whenever the aim of the enzymological study on AR is the *in vivo* inhibition of glucose reduction, then glucose itself, possibly combined with GAL, should be used to screen the ARDI potency *in vitro*. Similarly, when looking for molecules that can differentially inhibit GS-HNE reduction with respect to HNE reduction, GS-HNE itself should be used as a substrate of AR.

Finally, we firmly believe that it is time to switch from ARIs to ARDIs. In fact, ARDIs could be valuable for investigating AR-based useful/deleterious mechanisms and possibly for clarifying the relative impact of the double-sided action of this enzyme in experimental models. We also propose that a new strategic approach to controlling enzyme activity against diabetic complications and/or inflammation could be adopted.

## Supporting Information

Figure S1 **Enzymatic titration of HNE and GS-HNE.**
(DOCX)

Figure S2 **Effect of pH on the reduction of HNE and GS-HNE catalyzed by AR.**
(DOCX)

Figure S3 **Selwyn test for AR acting on HNE and GAL as substrates.**
(DOCX)

Figure S4 **Inhibition models of compound 18 on the AR dependent reduction of different substrates.**
(DOCX)

Figure S5 **Inhibition models of compound 19 on the AR dependent reduction of different substrates.**
(DOCX)

Protocol S1 **Synthesis of AR inhibitors.**
(DOCX)

Table S1 **Compounds Tested as Differential Aldose Reductase Inhibitors.**
(PDF)

Table S2 **Compounds Tested as Differential Aldose Reductase Inhibitors.**
(PDF)
