# Supplementary material for: A New Approach to Control the Enigmatic Activity of Aldose Reductase
Source: PLoS One. 2013 Sep 3;8(9):e74076. doi: 10.1371/journal.pone.0074076 (PMC3760808; doi:10.1371/journal.pone.0074076)
Supplement: Figure S1 — Enzymatic titration of HNE and GS-HNE. (DOCX) [file pone.0074076.s001.docx]

**Figure S1** **- Enzymatic titration of HNE and GS-HNE.**


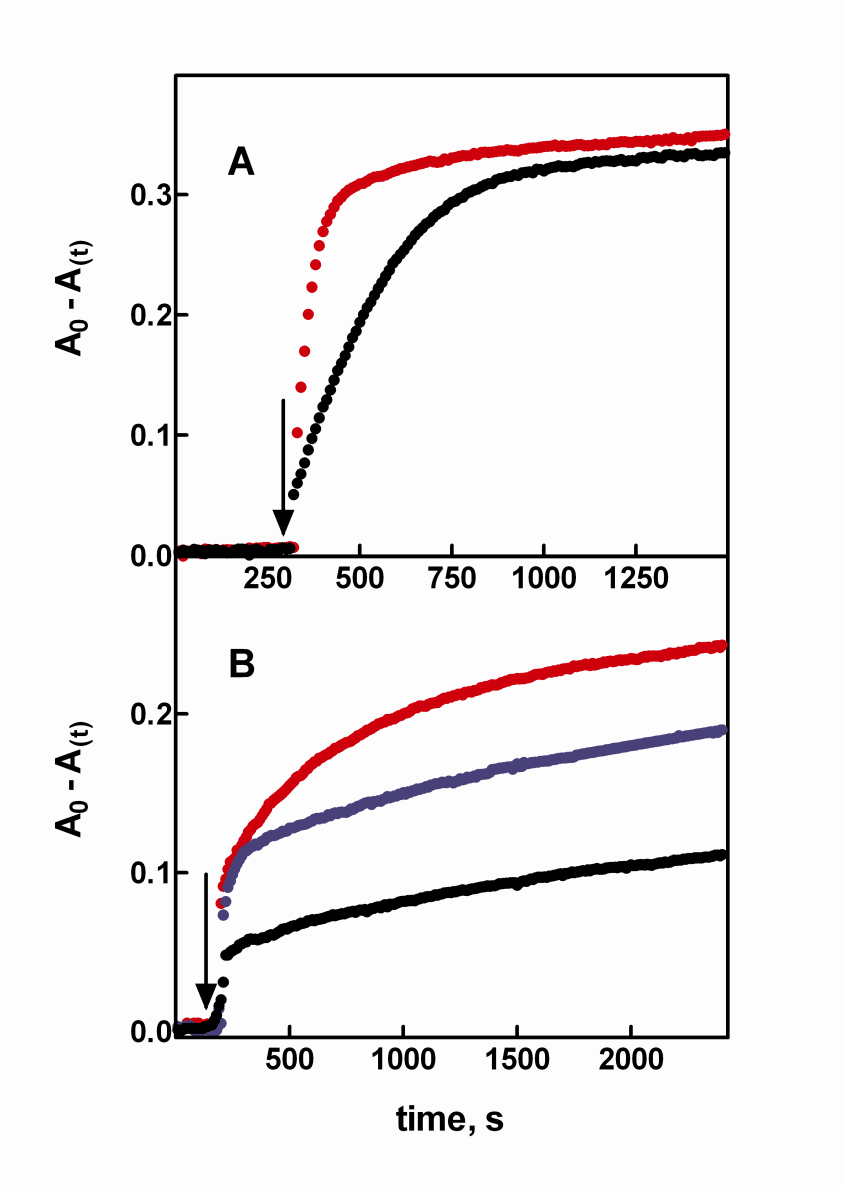


Solutions of HNE and GS-HNE were titrated through their reduction to the corresponding alchools catalyzed by AR, monitoring the decrease in absorbance at 340 nm associated to the NADPH oxidation (6.22 mM^-1^cm^-1^ extinction coefficient). The product concentration was determined by the difference of the absorbance measured at zero time (A_0_) and the absorbance measured at each time corrected for the NADPH oxidation occurring in the absence of substrates (A_t_). The latter reaction was considered as a first order kinetic reaction with a kinetic constant of 0.0048 min^-1^ (data not shown). *Panel A:* a 50 µM HNE solution, as measured by colorimetric assay (Gerard-Monnier et al. 1998. Chem. Res. Toxicol. 11, 1176), was incubated in the standard assay conditions at 37°C in the presence of either 8 mU (black curve) or 40 mU (red curve) of AR. The absorbance measured after 25 min accounted for a HNE concentration of 55 µM. *Panel B:* a 40 µM GS-HNE solution, as measured by colorimetric assay (Gerard-Monnier et al. 1998. Chem. Res. Toxicol. 11, 1176), was incubated in the standard assay conditions at 37 °C in the presence of 8 mU (black curve), 40 mU (blue curve) and 160 mU (red curve) of AR. The absorbance measured after 40 min in the presence the highest enzyme concentration (red curve) accounted for a GS-HNE concentration of 39 µM.

It is worth noting the higher amount of AR, with respect to the HNE titration, required to measure in a reasonable time the concentration of GS-HNE. This is consistent with the equilibrium to be displaced between GS-HNE hemiacetal and the free aldehyde.
