# Supplementary material for: A New Approach to Control the Enigmatic Activity of Aldose Reductase
Source: PLoS One. 2013 Sep 3;8(9):e74076. doi: 10.1371/journal.pone.0074076 (PMC3760808; doi:10.1371/journal.pone.0074076)
Supplement: Figure S2 — Effect of pH on the reduction of HNE and GS-HNE catalyzed by AR. (DOCX) [file pone.0074076.s002.docx]

**Figure S2 - Effect of pH on the reduction of HNE and GS-HNE catalyzed by AR.**


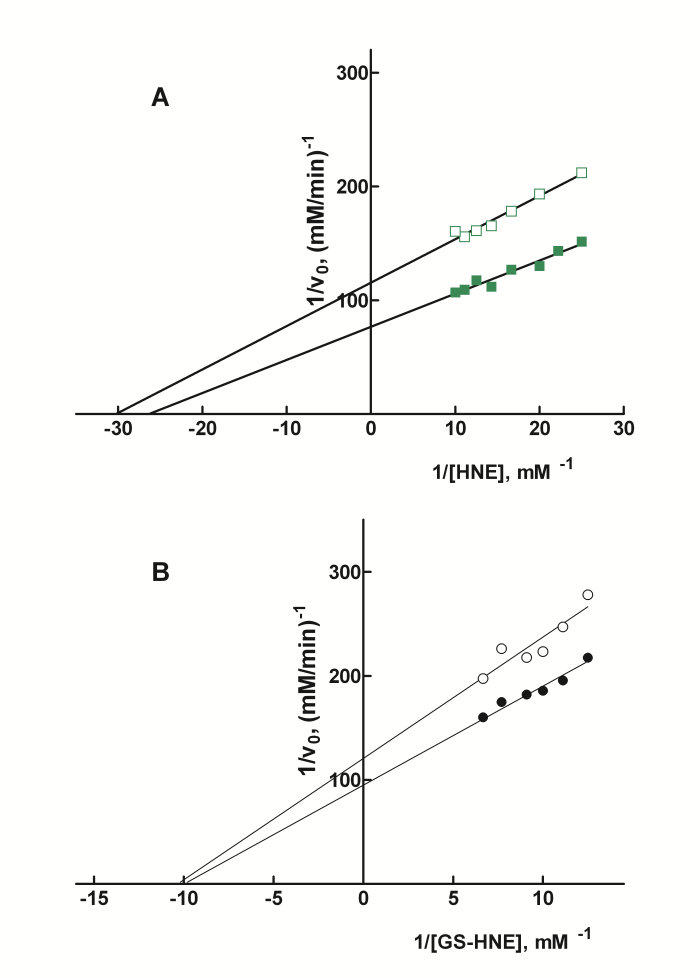


Double reciprocal plots of initial rate measurements of the AR catalyzed reduction of HNE (*Panel A*) and GS-HNE (*Panel B*). The assay was performed in standard conditions using 8 mU of purified AR at pH 6.8 (closed symbols) and pH 6.0 (open symbols).
