# Supplementary material for: A New Approach to Control the Enigmatic Activity of Aldose Reductase
Source: PLoS One. 2013 Sep 3;8(9):e74076. doi: 10.1371/journal.pone.0074076 (PMC3760808; doi:10.1371/journal.pone.0074076)
Supplement: Figure S3 — Selwyn test for AR acting on HNE and GAL as substrates. (DOCX) [file pone.0074076.s003.docx]

**Figure S3 - Selwyn test for AR acting on HNE and GAL as substrates.**


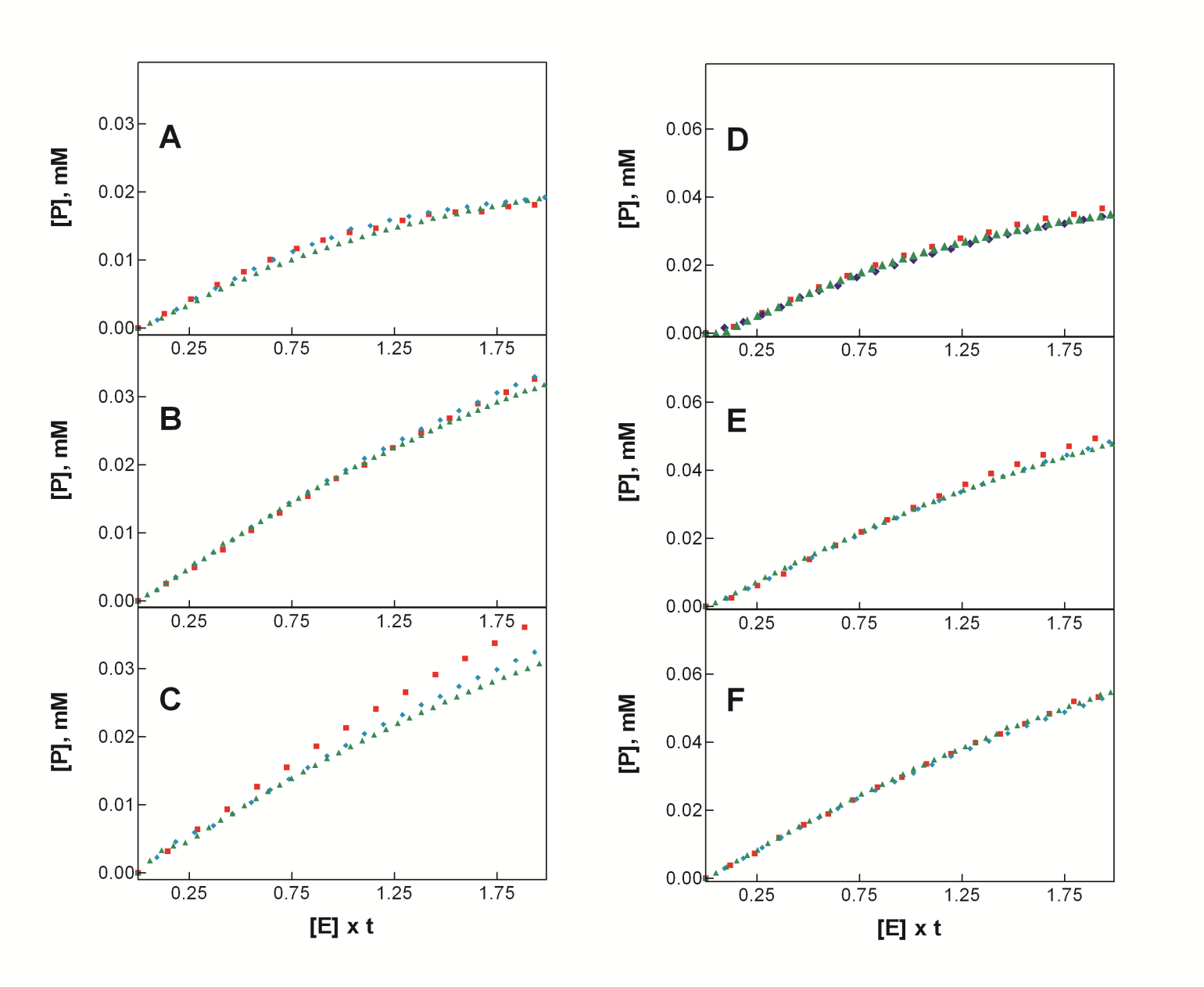


The AR catalyzed reaction was subjected to a Selwyn test using as substrates either HNE at a final concentration of 50, 110, 220 µM (Panels A, B and C, respectively) or GAL at a final concentration of 60, 110, 220 µM (Panels D, E and F, respectively).

In each panel the three curves refer to 3.5 mU (triangles), 5.6 mU (diamonds) and 8.8 mU (squares) of purified AR. The reaction was monitored following the decrease of absorbance at 340 nm linked to NADPH oxidation (extinction coefficient of 6.22 mM^-1^cm^-1^). The product concentration at different times was determined by the difference of the absorbance measured at zero time and the absorbance measured at each time corrected for the NADPH oxidation occurring in the absence of substrates. The latter reaction was considered as a first order kinetic reaction with a kinetic constant of 0.0048 min^-1^ (data not shown). The enzyme concentration was calculated on the basis of a molecular weight of 34 KDa.
