# Supplementary material for: A New Approach to Control the Enigmatic Activity of Aldose Reductase
Source: PLoS One. 2013 Sep 3;8(9):e74076. doi: 10.1371/journal.pone.0074076 (PMC3760808; doi:10.1371/journal.pone.0074076)
Supplement: Figure S5 — Inhibition models of compound 19 on the AR dependent reduction of different substrates. (DOCX) [file pone.0074076.s005.docx]

**Figure S5 - Inhibition models of compound 19 on the AR dependent reduction of different substrates.**


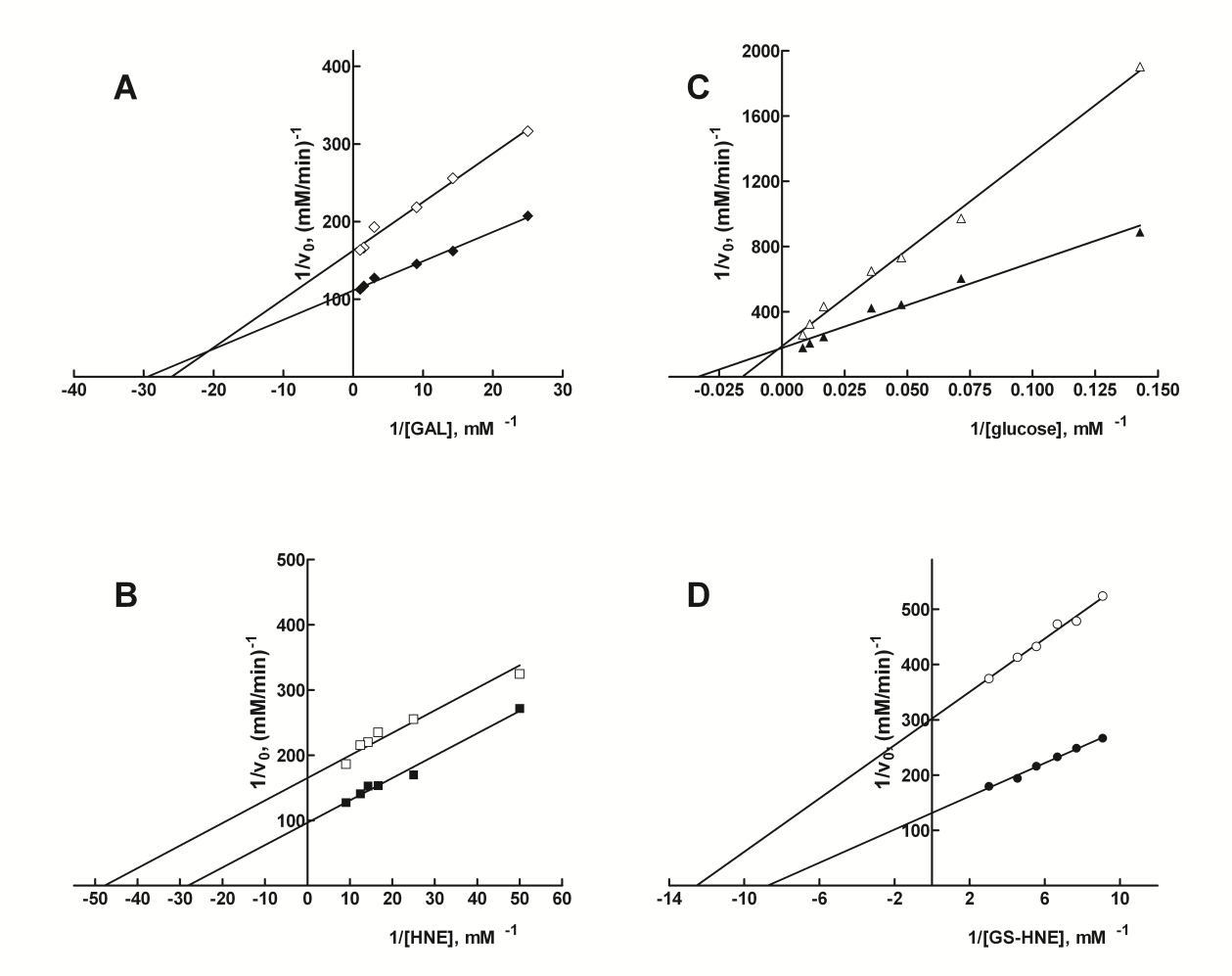


Double reciprocal plots of initial rate measurements of the AR catalyzed reduction of different substrates either in the absence (closed symbols) or in the presence (open symbols) of 47 µM of compound **19**. The assay was performed in standard conditions using approximately 8 mU of purified AR.
