# Supplementary material for: A New Approach to Control the Enigmatic Activity of Aldose Reductase
Source: PLoS One. 2013 Sep 3;8(9):e74076. doi: 10.1371/journal.pone.0074076 (PMC3760808; doi:10.1371/journal.pone.0074076)
Supplement: Protocol S1 — Synthesis of AR inhibitors. (DOCX) [file pone.0074076.s006.docx]

**Protocol S1**

**Synthesis of AR inhibitors**

**Chemistry.** Melting points were determined using a Reichert Köfler hot-stage apparatus and are uncorrected. Routine nuclear magnetic resonance spectra were recorded in DMSO-d_6_ solution on a Varian Gemini 200 spectrometer operating at 200 MHz. Mass spectra were obtained on a Hewlett-Packard 5988 A spectrometer using a direct injection probe and an electron beam energy of 70 eV.

Optical rotations were collected on a Perkin-Elmer 241 Polarimeter at 20±2 °C in water. Evaporations were performed in vacuo (rotary evaporator). Analytical TLCs were carried out on Merck 0.2 mm precoated silica gel aluminium sheets (60 F-254) and on Merk RP-18 glass plates (RP-18 F-254s). Flash Master Personal from Biotage was used as chromatographic system, with Snap Cartridges KP-C18-HS or KP-Sil from Biotage as columns.

**Synthesis of 4-substituted-4,7-dihydro-7-oxopyrazolo[1,5-*a*]pyrimidine-6-carboxylic acids (compounds 18 and 19).** The commercially available 3-aminopyrazole (10.0 mmol) was dissolved in 20 mL of glacial acetic acid. Diethyl ethoxymethylenemalonate (2.42 mL, 12.0 mmol) was then added and the resulting mixture was refluxed under stirring until the disappearance of the starting material (TLC analysis). The so obtained ethyl 4,7-dihydro-7-oxopyrazolo[1,5-*a*]pyrimidine-6-carboxylate, separated as a white solid, was collected by filtration, suspended in 5 mL of DMF and allowed to react, at 90 °C, with equimolar amounts of the suitable arylalkyl halide and anhydrous K_2_CO_3_ until the disappearance of the starting materials (TLC analysis). The solvent was then removed under reduced pressure and the resulting crude material was treated with crushed ice. The 4-substituted ester, separated as a pale yellow solid, was collected by filtration and hydrolyzed to the corresponding carboxylic acid through reaction with 5% NaOH, at 100° C, followed by acidification with concentrated HCl, under ice-cooling. The desired acid was collected, purified by crystallization from MeOH and characterized through physico-chemical and spectroscopic data.

Compound **18**: Yield: 74%. M.p. 242-245 °C. ^1^H-NMR (δ, ppm): 12.70 (s, 1H, COOH, exc.), 9.01 (s, 1H, H_5_), 8.02 (d, 1H, H_2_), 7.42-7.35 (m, 5H, ArH), 6.54 (d, 1H, H_3_), 5.49 (s, 2H, CH_2_).

Compound **19**: Yield: 26%. M.p. 260°C dec. ^1^H-NMR (δ, ppm): 9.13 (s, 1H, H_5_), 8.40 (s, 1H, H_2’_), 8.17 (d, 1H, H_4’_), 8.00 (d, 1H, H_2_), 7.87 (d, 1H, H_6’_), 7.66 (t, 1H, H_5’_), 6.56 (d, 1H, H_3_), 5.63 (s, 2H, CH_2_).

**Synthesis of 2-(5-methyl-7-oxo-4-phenethyl-4,7-dihydropyrazolo[1,5-a]pyrimidin-6-yl)acetic acid (compound 20)**. The commercially available 3-aminopyrazole (10.0 mmol) was dissolved in 20 mL of glacial acetic acid. Diethyl acetylsuccinate (2.40 mL, 12.0 mmol) was then added and the resulting mixture was refluxed under stirring until the disappearance of the starting material (TLC analysis). The so obtained ethyl 2-(4,7-dihydro-5-methyl-7-oxopyrazolo[1,5-*a*]pyrimidin-6-yl)acetate, separated as a white solid, was collected by filtration, suspended in 5 mL of DMF and allowed to react, at 90 °C, with equimolar amounts of the suitable arylalkyl halide and anhydrous K_2_CO_3_ until the disappearance of the starting materials (TLC analysis). The solvent was then removed under reduced pressure and the resulting crude material was treated with crushed ice. The 4-substituted ester, separated as a pale yellow solid, was collected by filtration and hydrolyzed to the corresponding carboxylic acid through reaction with 5% NaOH, at 100° C, followed by acidification with concentrated HCl, under ice-cooling. The desired acid was collected, purified by crystallization from EtOH and characterized by physico-chemical and spectroscopic data.

Yield: 35%, m.p. 275-277 °C. ^1^H-NMR: 7.92 (s, 1H, H_2_), 7.28 (s, 5H, ArH), 6.45 (s, 1H, H_3_), 4.32 (t, 2H, CH_2_), 3.55 (s, 2H, CH_2_), 3.02 (t, 2H, CH_2_), 2.27 (s, 3H, CH_3_)

**Synthesis of D-glyceramide and L-glyceramide (compounds 21 and 22)** . The suitable D- or L-glyceric acid calcium salt dihydrate (10.0 mmol), was converted to the corresponding acid by treatment with equimolar amount of HCl 0.1 N. Water was evaporated in vacuo and the resulting residue was diluted with methanol, filtered to remove salt, and allowed to react with trimethylsylil diazomethane, added dropwise until the persistence of a yellow colour, to obtain the key intermediate methyl D- or L-glycerate (TLC analysis). The solvent was then evaporated to dryness and the residue was diluted with 15.0 mL of 30% ammonium hydroxide and left under stirring at room temperature until the disappearance of the starting materials (TLC analysis). The solvent was then removed under reduced pressure and the resulting residue was diluted with methanol, filtered, and evaporated to dryness to obtain the target amide as a colorless syrup, which was purified by column chromatography (KP-C18-HS cartridge, CH_2_Cl_2_:MeOH = 9:1 as the eluant) and characterized by physico-chemical and spectroscopic data.

**D-Glyceramide**. Colourless syrup. Yield: 40%. [α]_D_: +14.8 (c=10%, H_2_O). ^1^H-NMR (δ, ppm): 7.15 (d, 2H, NH_2_, exc.), 3.81-3.75 (m, 2H, CH_2_), 3.55-3.39 (m, 1H, CH). *m/z*= [M^+^] 105 (12), 55 (100).

**L-Glyceramide**. Colourless syrup. Yield: 54%. [α]_D_: -12.5 (c=10.4, H_2_O). ^1^H-NMR (δ, ppm): 7.17 (d, 2H, NH_2_, exc.), 3.80-3.77 (m, 2H, CH_2_), 3.59-3.39 (m, 1H, CH). *m/z*= [M^+^] 105 (14), 55 (100).

**Synthesis of L-erythronamide, D-arabinonamide and L-arabinonamide (compounds 23, 24 and 25).** The suitable commercially available L-erythrose, D-arabinose or L-arabinose (10.0 mmol) was dissolved in 5 mL of water. Bromine (20.0 mmol) was added dropwise and the resulting orange solution was left overnight under stirring at room temperature. The excess of bromine was then quenched with 2% Na_2_S_2_O_3_ and the solution was evaporated to dryness. The residue obtained was diluted with dry methanol, filtered, and concentrated under vacuum to a colorless syrup, which was allowed to react with 30% ammonium hydroxide (10.0 mL) until the disappearance of the starting material (TLC analysis). The reaction mixture was evaporated to dryness and the resulting residue was washed with dry methanol, filtered, and evaporated under reduced pressure. The so obtained amide was purified by column chromatography (KP-C18-HS cartridge, CH_2_Cl_2_:MeOH = 9:1 as the eluant) and characterized by physico-chemical data.

**L-erythronamide**. Yield 35%. M.p. 97-100 °C (Lit.^1^ 91-92 °C). **D-arabinonamide**. Yield 35%. M.p.125-127 °C (Lit.^2^: 138-139 °C). **L-arabinonamide**. Yield 34%. M.p. 100-101 °C (Lit.^3^ 132-133 °C).
