# Supplementary material for: A New Approach to Control the Enigmatic Activity of Aldose Reductase
Source: PLoS One. 2013 Sep 3;8(9):e74076. doi: 10.1371/journal.pone.0074076 (PMC3760808; doi:10.1371/journal.pone.0074076)
Supplement: Table S1 — Compounds Tested as Differential Aldose Reductase Inhibitors. (PDF) [file pone.0074076.s007.pdf]

Table S1. Compounds Tested as Differential Aldose Reductase Inhibitors.

**1**	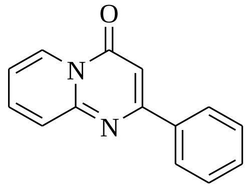	**2**	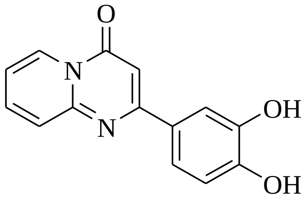	**3**	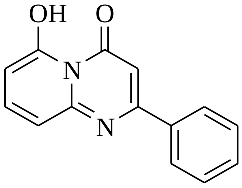
**4**	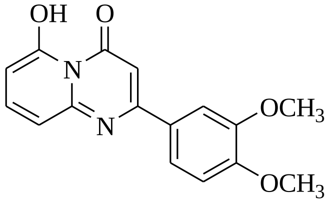	**5**	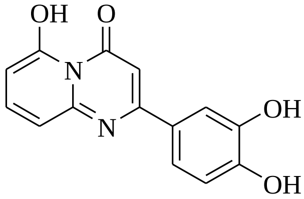	**6**	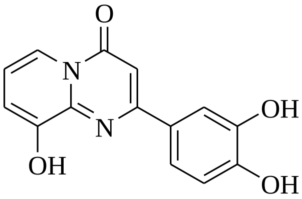
**7**	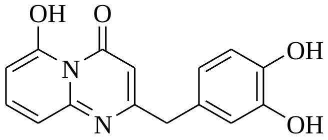	**8**	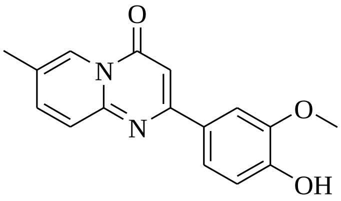	**9**	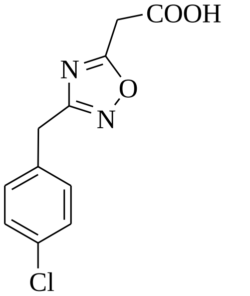
**10**	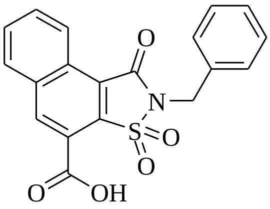	**11**	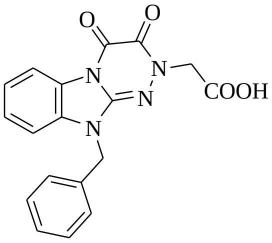	**12**	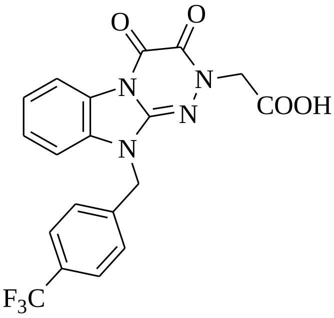
**13**	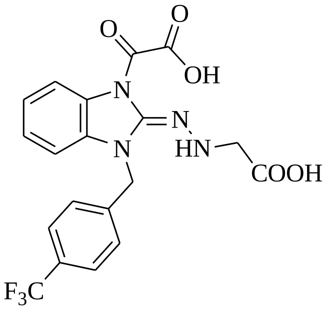	**14**	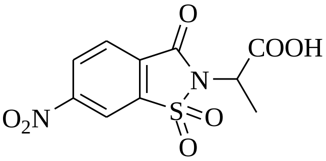	**15**	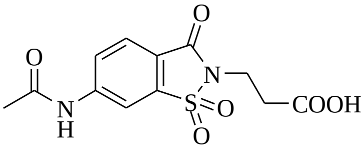
**16**	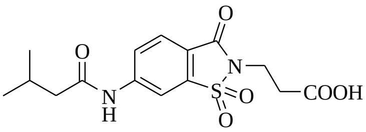	**17**	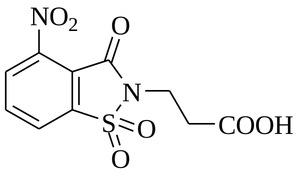	**18**	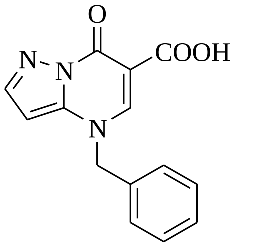
**19**	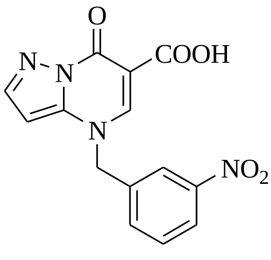	**20**	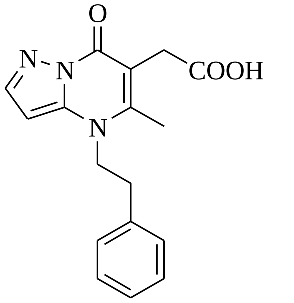		
