# Supplementary material for: A New Approach to Control the Enigmatic Activity of Aldose Reductase
Source: PLoS One. 2013 Sep 3;8(9):e74076. doi: 10.1371/journal.pone.0074076 (PMC3760808; doi:10.1371/journal.pone.0074076)
Supplement: Table S2 — Compounds Tested as Differential Aldose Reductase Inhibitors. (PDF) [file pone.0074076.s008.pdf]

Table S2. Compounds Tested as Differential Aldose Reductase Inhibitors.

**21**	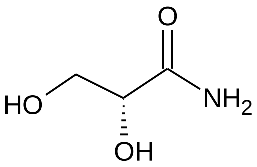	**22**	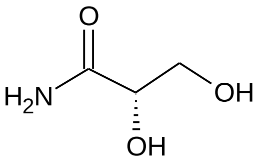	**23**	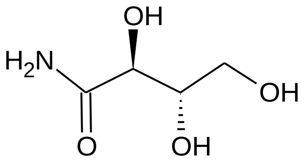
**24**	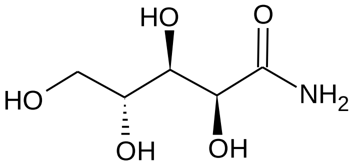	**25**	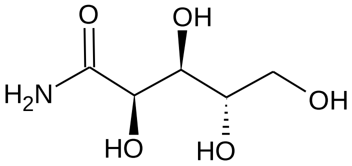	**26**	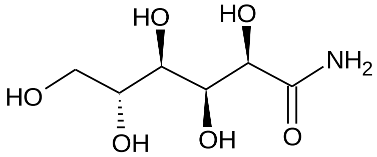
**27**	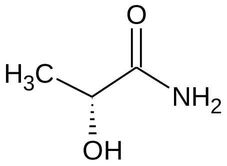	**28**	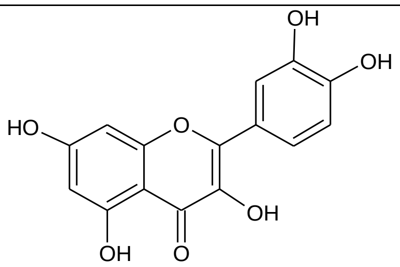	**29**	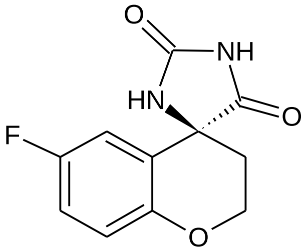
**30**	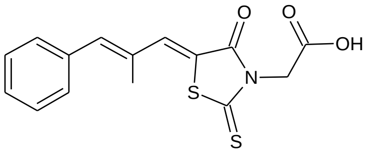				
